# Identification of RSV Fusion Protein Interaction Domains on the Virus Receptor, Nucleolin

**DOI:** 10.3390/v13020261

**Published:** 2021-02-08

**Authors:** Peter Mastrangelo, Allysia A. Chin, Stephanie Tan, Amy H. Jeon, Cameron A. Ackerley, Karen K. Siu, Jeffrey E. Lee, Richard G. Hegele

**Affiliations:** 1 Department of Laboratory Medicine and Pathobiology, University of Toronto, Toronto, ON M5S 1A8, Canada; peter.mastrangelo@utoronto.ca (P.M.); allysia.chin@utoronto.ca (A.A.C.); stephanie.lj.tan@gmail.com (S.T.); amy.jeon@kpu.ca (A.H.J.); cameron.ackerley@gmail.com (C.A.A.); karen.siu@utoronto.ca (K.K.S.); jeff.lee@utoronto.ca (J.E.L.); 2 Department of Paediatric Laboratory Medicine, The Hospital for Sick Children, Toronto, ON M5G 1X8, Canada

**Keywords:** RSV, nucleolin, fusion protein, protein–protein interactions

## Abstract

Nucleolin is an essential cellular receptor to human respiratory syncytial virus (RSV). Pharmacological targeting of the nucleolin RNA binding domain RBD1,2 can inhibit RSV infections in vitro and in vivo; however, the site(s) on RBD1,2 which interact with RSV are not known. We undertook a series of experiments designed to: document RSV-nucleolin co-localization on the surface of polarized MDCK cells using immunogold electron microscopy, to identify domains on nucleolin that physically interact with RSV using biochemical methods and determine their biological effects on RSV infection in vitro, and to carry out structural analysis toward informing future RSV drug development. Results of immunogold transmission and scanning electron microscopy showed RSV-nucleolin co-localization on the cell surface, as would be expected for a viral receptor. RSV, through its fusion protein (RSV-F), physically interacts with RBD1,2 and these interactions can be competitively inhibited by treatment with Palivizumab or recombinant RBD1,2. Treatment with synthetic peptides derived from two 12-mer domains of RBD1,2 inhibited RSV infection in vitro, with structural analysis suggesting these domains are potentially feasible for targeting in drug development. In conclusion, the identification and characterization of domains of nucleolin that interact with RSV provide the essential groundwork toward informing design of novel nucleolin-targeting compounds in RSV drug development.

## 1. Introduction

According to the World Health Organization (WHO), respiratory syncytial virus (RSV), or human orthopneumovirus, is a leading cause of acute lower respiratory infection and hospitalization in infants and young children [1]. Currently, there are few options for prophylaxis and therapy and those available are largely reserved for select clinical populations in high-resource healthcare settings. While there are numerous ongoing efforts to develop an RSV vaccine, it has proven difficult. After almost 60 years of research, no vaccine is currently available [2]. In this time of the COVID-19 pandemic, the possibility looms that we may, in the coming flu season, have to deal with a resurgence of COVID-19, the influenza virus, and RSV simultaneously [3]. Given this, it is even more critical that novel therapeutics be developed to deal with RSV. In 2011, our group identified cell surface nucleolin as an essential receptor for RSV infection [4]. In this work, we showed that interaction of nucleolin with RSV, in particular the RSV fusion protein (RSV-F), was required for infection, and when nucleolin was specifically silenced via siRNA, RSV infection was reduced. In subsequent studies, we showed that targeting nucleolin with the DNA aptamer, AS1411, originally developed as a cancer therapeutic, could not only reduce infection of cells in culture, but in two animal models with a corresponding reduction of lung inflammation [5]. Recently, it has also been shown that targeting a pathway that controls shuttling of nucleolin to the cell surface is a viable strategy to reduce RSV infection [6].

Human nucleolin is a 707 a.a. multifunctional phosphoprotein that is composed of three main structural domains: the N-terminal domain, which is highly acidic and contains phosphorylation sites, the central domain, which contains four RNA binding domains (RBDs), and the C-terminal domain, which includes a nucleolar localization signal and a GAR/RGG domain involved in protein binding [7,8]. While primarily found in the nucleus and cytoplasm of the cell, a small percentage of the protein is located on the cell surface where it serves as a binding partner for molecules involved in a range of functions including cell differentiation and tumorigenesis [8]. Cell surface nucleolin has also been shown to play a role in viral attachment or entry for a number of viruses in addition to RSV. These include EV71 [9], HIV [10], multiple influenza A viruses such as H1N1, H3N2, H5N1, and H7N9 [11], as well as PIV3 [12].

RSV attachment and entry involves multiple proteins. Attachment can occur through the viral G protein or the F protein. While attachment via the G protein is not absolutely required, as shown by the ability of ΔG RSV to infect cells [13], it does greatly improve the efficiency of infection and likely involves CX3CR1 [14]. Attachment and viral entry through RSV-F involve proteins such as IGFR1 and nucleolin [4,6].

Here we present the results of a series of experiments designed to look more closely into the molecular details of the interactions between nucleolin and RSV. First, we confirmed co-localization of nucleolin and RSV on the cell surface at the ultrastructural level by using immunogold electron microscopy. This was followed by a series of biochemical experiments where we identified domains in which nucleolin physically interacts with RSV, followed by confirmation of these physical interactions in living systems using functional in vitro RSV infection assays. Finally, we carried out structural analysis to further characterize aspects of these interaction domains with the goal of informing future drug development.

## 2. Materials and Methods

### 2.1. Electron Microscopy

#### 2.1.1. Culture of MDCK Cells and RSV Infection for Electron Microscopy

MDCK cells were cultured in EMEM (Thermo Fisher Scientific, Waltham, MA, USA) supplemented with 10% fetal bovine serum (FBS; *v*/*v*) (Thermo Fisher Scientific, Waltham, MA, USA) and antibiotics. The next day, RSV A2 (ATCC, Manassas, VA, USA), prepared by centrifugation as previously described [15], was added and incubated for one hour at 37 °C and 5% CO_2_ and collected and fixed for EM. Cells that had no viral exposure were also collected after one hour as a control.

#### 2.1.2. Routine Transmission Electron Microscopy

Portions of membranes from all treatment groups were fixed in 2.5% glutaraldehyde (*w*/*v*) in 0.1 M phosphate buffer, pH 7.2. They were then rinsed in buffer and postfixed in 1% OsO_4_ (*w*/*v*) for an additional hour. Samples were dehydrated in an ascending series of acetone and infiltrated in Embed 812-Araldite. Samples were then flat embedded in Embed 812-Araldite and polymerized overnight at 60 °C. Cross sections of the samples were then cut with a diamond knife and an ultramicrotome and mounted on grids. Ultrathin sections were stained with 2% uranyl acetate (*w*/*v*) and lead citrate prior to imaging in a JEOL JEM 1011 transmission electron microscope (JEOL USA, Peabody, MA, USA) (TEM).

#### 2.1.3. Cryo-Ultramicrotomy, Immunogold Labeling, and Cryo-Section Embedding

Portions of cell pellets or cells on membranes were fixed in 4% paraformaldehyde (*w*/*v*) and 0.1% glutaraldehyde (*w*/*v*) in 0.1 M phosphate buffer. Sheets of epithelial cells were removed from the fixed membranes with a sharp, single edge razor blade and placed in Eppendorf tubes containing buffer. The sheets of cells were then embedded in gelatin and cryoprotected with 2.3 M gelatin. The samples were then frozen on Al microtomy pins and frozen in liquid nitrogen prior to being sectioned with a diamond knife at −120 °C in a cryo-ultramicrotome. The samples were then transferred to formvar-coated nickel grids in a loop of molten sucrose. The grids with ultrathin frozen sections of cross sectioned MDCK cells were then double labeled using immunogold for RSV and nucleolin. Grids were washed in PBS containing 5% BSA (*w*/*v*) and 0.15% glycine (*w*/*v*), followed by two 5 min washes in PBS/BSA. The grids were then incubated in a monoclonal antibody against RSV (NB100-65217; Novus Biologicals, Toronto, ON, Canada) and diluted 1:5 in PBS/BSA for an hour. They were then rinsed thoroughly in PBS/BSA and incubated in 5 nm gold goat anti-murine IgG complexes for another hour. They were then rinsed thoroughly in PBS/BSA and the process was repeated using a polyclonal antibody against nucleolin (H-250; Santa Cruz Biotechnology, Dallas, TX, USA) and diluted 1:100 in PBS/BSA and 15 nm gold goat anti-rabbit complexes. Following PBS/BSA washes, the grids were placed face down on drops of 0.5% OsO_4_ (*w*/*v*) in 0.1 M phosphate buffer (pH 7.2) on a sheet of Parafilm for 10 min, then washed on drops of 7% sucrose (*w*/*v*), and post-stained for 20 min on a drop of 0.5% uranyl acetate (*w*/*v*) in barbital/acetate buffer, pH 5.2, containing 5% sucrose (*w*/*v*). The grids were then placed section side down on drops of ethanol solutions from 40% to 95%, followed by 100% ethanol (*v*/*v*) for 2 min at each step, and finally infiltrated with 2% Embed 812-Araldite in ethanol (*w*/*v*). After infiltration, the grids were blotted between two disks of hardened filter paper to remove excess monomer and subsequently polymerized overnight in a vacuum oven at 60 °C prior to examination in the TEM [16].

#### 2.1.4. Routine Scanning Electron Microscopy

Portions of membranes were fixed in 2.5% glutaraldehyde (*w*/*v*) in 0.1 M phosphate buffer, pH 7.2. They were then rinsed in buffer and postfixed in 1% OsO4 (*w*/*v*) for an additional hour. Samples were dehydrated in an ascending series of ethanol washes and critical point dried, after which specimens were mounted on Al stubs with carbon paint and rendered conductive with a thin layer of sputter-coated Pt. Finally, they were examined in a JEOL JSM 6700F field emission scanning electron microscope (JEOL USA, Peabody, MA, USA) and images were recorded.

#### 2.1.5. Immunogold Surface Labeling

As with the immunogold labeled TEM samples, portions of membranes were fixed in 4% paraformaldehyde (*w*/*v*) and 0.1% glutaraldehyde (*w*/*v*) in 0.1 M phosphate buffer. They were then double labeled using immunogold for RSV and nucleolin in a similar fashion to the TEM samples. Membranes were washed in PBS containing 5% BSA (*w*/*v*) and 0.15% glycine (*w*/*v*), followed by two 5 min washes in PBS/BSA. The membranes were then incubated in a monoclonal antibody against RSV (NB100-65217; Novus Biologicals, Toronto, ON, Canada) and diluted 1:5 in PBS/BSA for an hour. They were then rinsed thoroughly in PBS/BSA and incubated in 5 nm gold goat anti-murine IgG complexes for another hour. They were then rinsed thoroughly in PBS/BSA and the process was repeated using a polyclonal antibody against nucleolin (H-250; Santa Cruz Biotechnology, Dallas, TX, USA) and diluted 1:100 in PBS/BSA and 15 nm gold goat anti-rabbit complexes. In both TEM and SEM immunocytochemical studies, controls included the omission of either one of the primary antibodies or either one of the gold samples. Samples were then critical point dried, mounted on Al stubs with carbon paint, and rendered conductive with a thin coat of evaporated carbon. They were then examined in a JEOL JSM 6700F field emission scanning SEM, using either a backscatter electron detector or a “Wein-type” filter [17], and images were recorded.

### 2.2. Protein Preparation and Characterization

The RBD1,2 domains of human nucleolin were expressed recombinantly with a C-terminal His tag and purified as described previously [18]. Briefly, RBD1,2 protein was expressed in a protease-deficient *E. coli* strain using the pET-21a vector system (The pET-21aRBD1,2 vector was a gift from Dr. Paula J. Bates, University of Louisville, KY). After growing cells in appropriate selective media and inducing protein expression, a clarified lysate was prepared and the protein was purified using nickel sepharose chromatography. The protein was further purified using size exclusion chromatography. Protein fractions were analyzed on 4–20% Tricine SDS-PAGE and silver stained to confirm purity.

### 2.3. Virus Immobilization with RBD1,2 Coated Beads

His Mag Sepharose^TM^ excel beads (GE Healthcare, Danderyd, Sweden) were equilibrated in a wash buffer (50 mM imidazole, 0.5 M NaCl, 20 mM phosphate buffer, pH 7.4). RBD1,2 was then bound to equilibrated beads in 1 M NaCl, 40 mM phosphate buffer, pH 7.4, for 30 min at room temperature and then washed twice and blocked with 1% skimmed milk in 1 M NaCl, 40 mM phosphate buffer, pH 7.4, and Complete^TM^ protease inhibitors (Roche, Mississauga, ON, Canada) for 20 min at room temperature. The beads were then washed one time in IP buffer (0.1% skimmed milk in 1 M NaCl, 40 mM phosphate buffer, pH 7.4, and protease inhibitors) and incubated in IP buffer with 1 × 10^6^ cfu of RSV with and without Palivizumab overnight at 4 °C. The next day, the beads were washed three times and protein eluted with 0.5 M imidazole in wash buffer. Eluates were added to Laemmli buffer and run on SDS-PAGE for Western blot analysis. The polyvalent mouse anti-RSV-F1 antibody (NCL-RSV3, Leica Biosystems, UK) and D6 mouse anti-nucleolin monoclonal (Santa Cruz Biotechnology, Dallas, TX), which also recognized RBD1,2, were used as primary antibodies (1:1000 dilution).

### 2.4. Virus Pre-Incubation with RBD1,2 before Infection of Tissue Culture Cells

The assay described is a variation on our previously published fluorescence focus assay [5]. Briefly, HEp-2 cells were cultured in EMEM supplemented with 10% fetal bovine serum (FBS; *v*/*v*), antibiotics, and plated onto black flat clear-bottomed 96 well plates (2.5 × 10^4^ cells/well). RSV was pre-incubated with different concentrations of the mini-protein (or an equal amount of a mock purification) for 3 h at 37 °C/5% CO_2_, then the mixture was put on HEp-2 cells in 96 well plates for 90 min. Cultures were washed twice with PBS to remove the unbound virus, fresh protein or control was added, and the cultures left overnight at 37 °C/5% CO_2_ to allow at least one full round of virus replication.

The cells were then fixed with 75% cold acetone in PBS for 5 min. Following three washes with PBS, 0.1% Tween-20 (PBS-Tween; *v*/*v*) cells were incubated with a 1:10 dilution of NCL-RSV3 in PBS-Tween, 10% FBS for 30 min at room temperature. The cells were then washed again as before and incubated for 30 min with 1:400 dilution (5 μg/mL) of Alexa Fluor 488 anti-mouse IgG and a 1:200 dilution of ToPro3 (Life Technologies, Thermo Fisher, Waltham, MA, USA) in PBS-Tween, 10% FBS (*v*/*v*). After a final washing, fluorescence was detected with a Tecan Infinite M200Pro^TM^ plate reader (ex. 488 nm, em. 519 nm).

In this assay, the quantity of infected cells per well is converted to a fluorescent signal. To assay cellular toxicity, the “Celltiter 96” cell proliferation assay (Promega, Madison, WI, USA) was performed according to manufacturer’s instructions.

### 2.5. Peptide Array Virus Overlay Protein Binding Assay (VOPBA)

Peptide array of the RBD1,2 protein, blotted on amine-functionalized cellulose membrane (12-mer peptides with 4 mer overlap), was purchased from PepMetrix Technology Ltd. (Richmond, BC, Canada). The membrane was activated with methanol and blocked with a membrane blocking buffer (MBS) containing Casein-based blocking buffer concentrate (Sigma-Aldrich, St. Louis, MO, USA) in TBS and 5% (*w*/*v*) sucrose at 4 °C overnight with gentle rocking. The membrane was then incubated with concentrated purified RSV A2 at room temperature for 3 h, followed by incubation with a primary virus-specific polyclonal antibody (Meridian Life Sciences, Cincinnati, OH, USA) at 4 °C overnight with gentle rocking, and with a secondary HRP-conjugated antibody (Abcam, Cambridge, UK) at room temperature for 1 h. The signal was visualized using the SuperSignal West Pico Chemiluminescent Substrate (Thermo Fisher Scientific, Waltham, MA, USA) and detected using the Fusion FX7 imaging system (Montreal Biotech Inc., Montreal, QC, Canada).

### 2.6. Protein Structural Illustrations and Electrostatic Potential Calculations

Structural illustrations of human nucleolin, RBD1,2 (PDB code: 2KRR; [18]), and RSV-F (PDB code: 6VKD; [19]) were generated using MacPyMOL (version 2.3.5; Schrodinger LLC) and Biorender. Electrostatic potentials were calculated using the APBS plugin in MacPyMOL at a salt concentration of 0.15 M.

## 3. Results

### 3.1. Transmission Electron Microscopy (TEM), Co-Localization of RSV and Nucleolin at the Cell Surface, and Scanning Electron Microscopy (SEM) Co-Localization of RSV and Nucleolin on the Cell Surface

**Figure 1:**
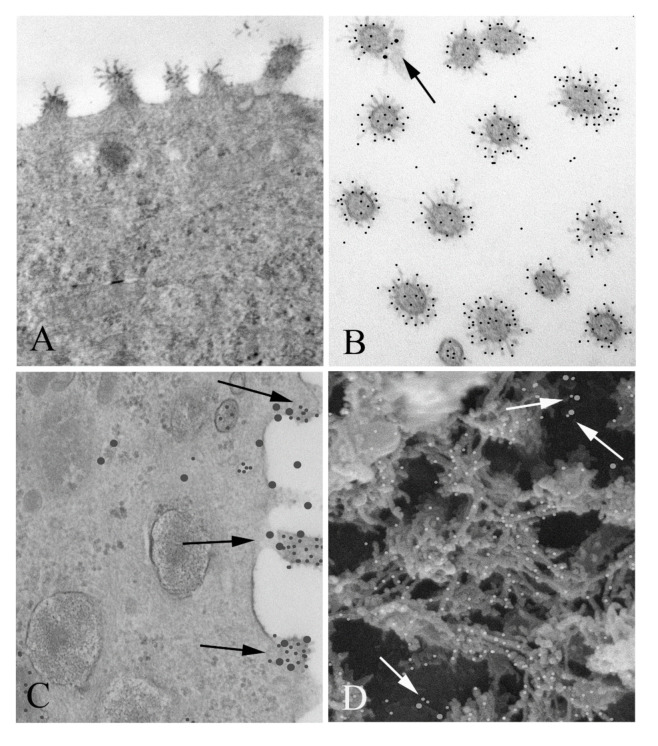
Co-localization of respiratory syncytial virus (RSV) and nucleolin in infected MDCK cells: (**A**) TEM of an RSV infected MDCK cell. Note the virus particles associated with the apical surface. Magnification 60,000×. (**B**) Cross sections of virus particles near the apical surface which have been double immunogold labeled for RSV (small particles) and nucleolin (large particles). A small portion of the apical membrane is seen with the virus, which is labeled with large particles (arrow). Magnification 60,000×. (**C**) TEM of a RSV infected MDCK cell, immunogold labeled for nucleolin and RSV. Nucleolin is seen at the periphery of the viral particles associated with the apical membrane (arrows) (large particles), while RSV antibody label (small particles) was confined to the interior of the virus. (**D**) SEM of the surface of an infected cell was imaged using a Wein filter. Numerous co-localizations are seen (arrows). Magnification 70,000×.

RSV-infected MDCK cells were prepared for TEM and then double labeled using immunogold for RSV and nucleolin to visualize co-localization at the cell surface, as expected for a viral receptor (Figure 1). MDCK cells were used for this study because they, like the airway epithelium of the lung, are polarized, and thus have distinct apical and basal ends [20]. As can be seen in Figure 1C, there are many instances of co-localization between RSV and nucleolin at the cell surface. SEM was also performed and then double labeled with immunogold as for TEM. It is striking that nucleolin virus co-localization is also apparent from the outside surface of the cell, clearly showing that nucleolin interacted with the virus before it entered the cell (Figure 1D). This result encouraged us to further characterize this interaction biochemically.

### 3.2. RSV Can Be Immobilized on Sepharose Beads Coated with RBD1,2 Domains of Nucleolin

**Figure 2:**
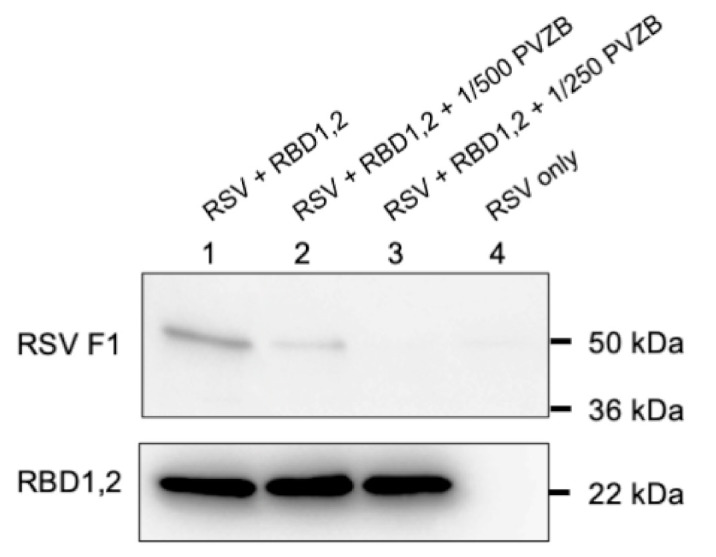
RSV co-immunoprecipitation by RBD1,2 is inhibited by Palivizumab. Adding increasing concentrations of the anti-RSV-F antibody Palivizumab inhibits this interaction (lanes 1, 2 and 3). Lane 4 is an RSV-only control. RSV-F1 protein is detected using NCL-RSV3 primary antibody and RBD1,2 with D6 anti-nucleolin monoclonal antibody. This experiment was repeated 4 times.

Previously, we have shown that RSV infection can be inhibited by the DNA aptamer, AS1411, in cell culture and in two different animal model systems [5]. AS1411 is known to bind to the RBD1,2 domains of human nucleolin [18], so we wanted to see if RBD1,2 could bind to RSV. In a modification of the standard immunoprecipitation assay, we prepared magnetic beads bound to recombinant RBD1,2 via an His tag and used them to immobilize intact virus. As a control, we co-incubated the RSV-F specific antibody Palivizumab to verify the specificity of the interaction (Figure 2). Not only did this show that RBD1,2 bound to RSV, but that this interaction went through the viral fusion protein, which is confirmed by the ability of Palivizumab to block it.

### 3.3. RBD1,2 Can Block Infection of HEp-2 Cells in Cell Culture

**Figure 3:**
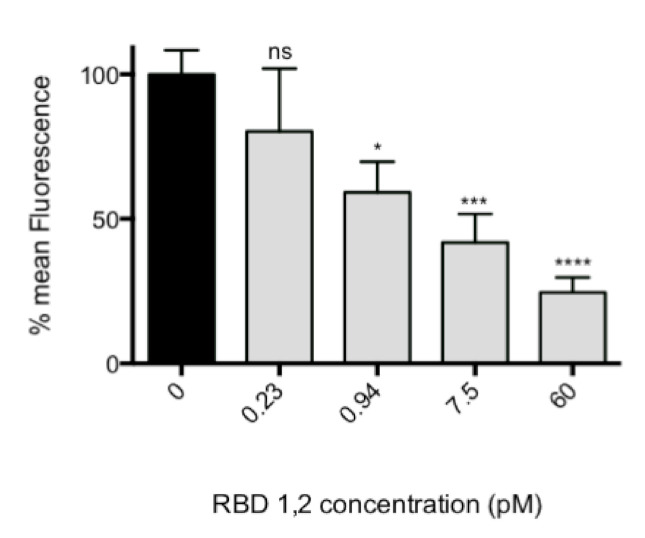
RBD1,2 vs. virus competition assay with HEp-2 cells, unattached virus removed. Note that 60 pM of mini-protein is sufficient to reduce virus replication by 80%. *p* < 0.0001, one-way ANOVA, average of 5 experiments. Bonferroni’s multiple comparison test. ns, not significant; *, *p* < 0.05; ***, *p* < 0.001; ****, *p* < 0.0001.

To confirm that the interaction of RBD1,2 and the viral fusion protein had biological relevance (i.e., were not interactions seen only in a biochemical assay, but also in living systems), we performed an infection assay of HEp-2 cells in which purified RSV was pre-incubated with RBD1,2 before viral challenge of HEp-2 cell cultures. A concentration of 60 pM of RBD1,2 was sufficient to block infection by 80% (*p* < 0.0001; Figure 3). There was no toxicity detected over the protein concentration range using the CellTiter96 cell proliferation assay (not shown).

### 3.4. Whole RSV Virus Probe of Nucleolin Peptide Arrays Reveal a Number of Potential Virus Interaction Sites

**Figure 4:**
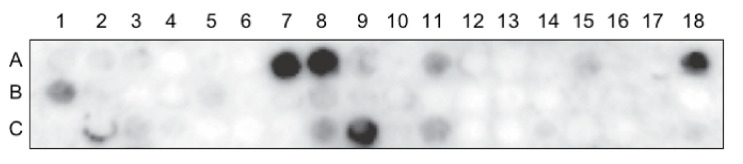
Peptide array of the entire RBD1,2 protein (a.a. 300–466 of human nucleolin) probed with RSV. Spot #A1 is in the top left corner reading from left to right across the array. This experiment was repeated 4 times.

In a complementary set of experiments to identify sites on nucleolin that physically interact with RSV, we employed peptide arrays (12-mer array, 4-mer overlap) that encompassed the whole RBD1,2 protein in a modification of our previously published VOPBA assay [4]. In Figure 4, an example of one of these nucleolin arrays is presented, showing interactions as dark spots. Four spots were found to appear consistently by giving off relatively strong signals (see Table 1) and were selected for further characterization in virus infection assays.

**Figure 5:**
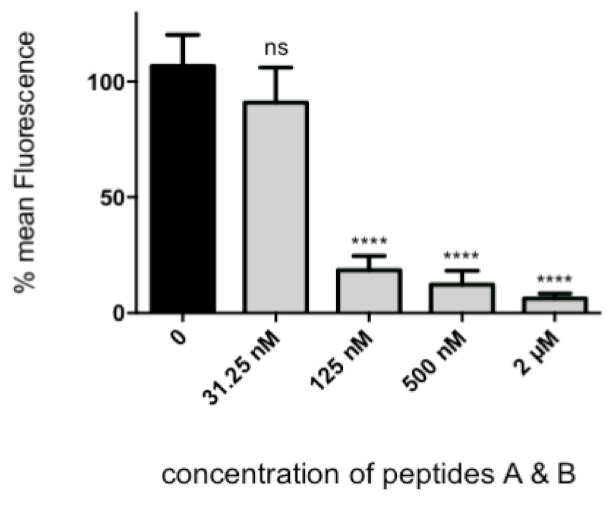
Equimolar quantities of peptide A and B vs. virus competition assay with HEp-2 cells. Note that 2 μM of peptides A and B are sufficient to reduce virus replication by 94%. *p* < 0.0001, average of 6 experiments. Bonferroni’s multiple comparison test. ns, not significant; ****, *p* < 0.0001.

**Figure 6:**
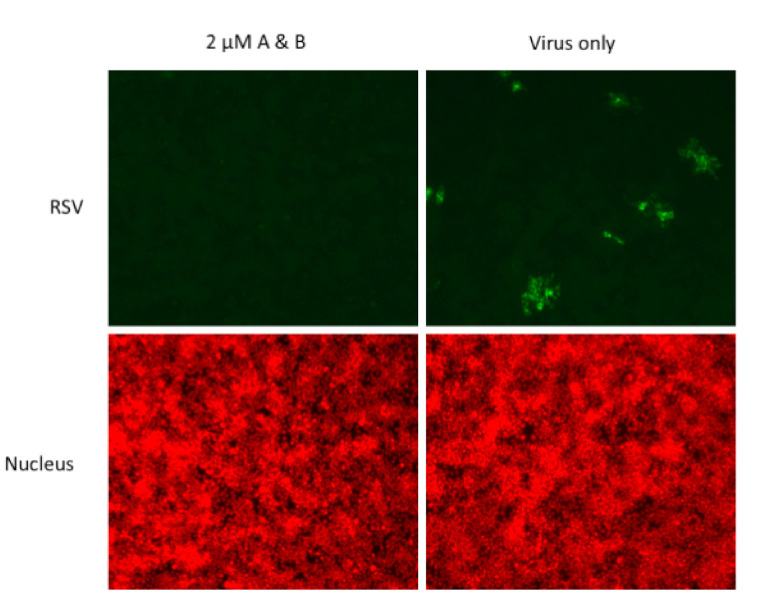
Fluorescence photographs from representative wells of HEp-2 cells immunostained for RSV and counterstained for nucleus as a control. Note the almost complete absence of RSV staining in the well treated with peptide A and B.

Two of the peptides, TEPTTAFNLFVG and MTRKFGYVDFES, henceforth called peptide A and B, respectively, had a modest amount of inhibitory activity when used individually. By contrast, when these peptides were combined in equimolar concentrations, significant inhibitory activity was observed in the nanomolar to micromolar range, with 2 μM of combined peptides reducing infection by 94% (*p* < 0.0001, Figure 5 and Figure 6). There was no toxicity detected over the protein concentration in this experiment using the CellTiter96 cell proliferation assay (not shown).

## 4. Discussion

**Figure 7:**
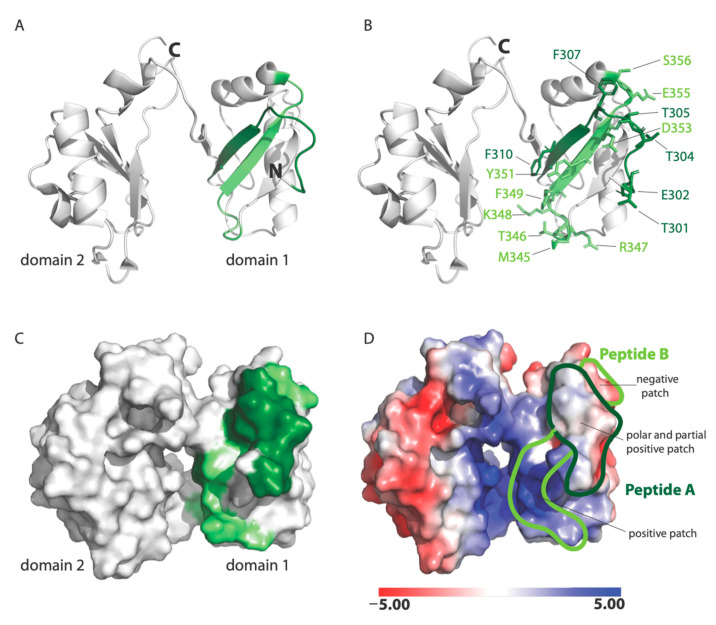
NMR solution structure of human nucleolin, RBD1,2 (PDB code: 2KRR): (**A**) Ribbon-structure representation showing peptide A in dark green and peptide B in light green. Note that the peptides are anti-parallel and are part of a *β*-sheet; (**B**) surface-exposed residues in peptides A and B are shown in stick format; (**C**) a surface representation of RBD1,2. Peptides A and B are colored in dark and light green, respectively; (**D**) The electrostatic potential representation of RBD1,2. Electrostatic potential units are in KT/e.

In this study, we have extended previous work of RSV–nucleolin interactions through ultrastructural confirmation of co-localization on the cell surface and show how recombinant proteins and standard biochemical assays, as well as peptide array technology, can be very useful in mapping protein–protein interactions when combined with regular verification of biological relevance using cell culture model systems. This work was done in MDCK and HEp-2 culture systems and will need to be further confirmed in human airway epithelial cultures, similarly to what has been done for studies of the RSV-G protein receptor CX3CR1 [14,21,22]. Our data show that RSV binds nucleolin through the RBD1,2 domain and more specifically involves two 12-mer stretches within RBD1,2 (Figure 7). It is fascinating that the peptides A and B both work cooperatively to block virus infection and also lie close to each other within two antiparallel strands of a β-sheet found in the RBD1 domain of nucleolin (Figure 7A; [18]). Peptides A and B form a contiguous surface, with many of its side chains exposed on the surface of the RBD1,2 protein and available for binding (Figure 7B). The surface-exposed residues of peptide A (Thr301, Glu302, Thr304, Thr305, Phe307, and Phe310) are predominantly aromatic or polar, with only one negatively charged residue. Peptide B is positively charged on one end and negatively charged on the other. Aromatic and polar residues (Met345, Thr346, Arg347, Lys348, Phe349, Tyr351, Asp353, Glu355, and Ser356) are also presented on the surface of peptide B (Figure 7B).

**Figure 8:**
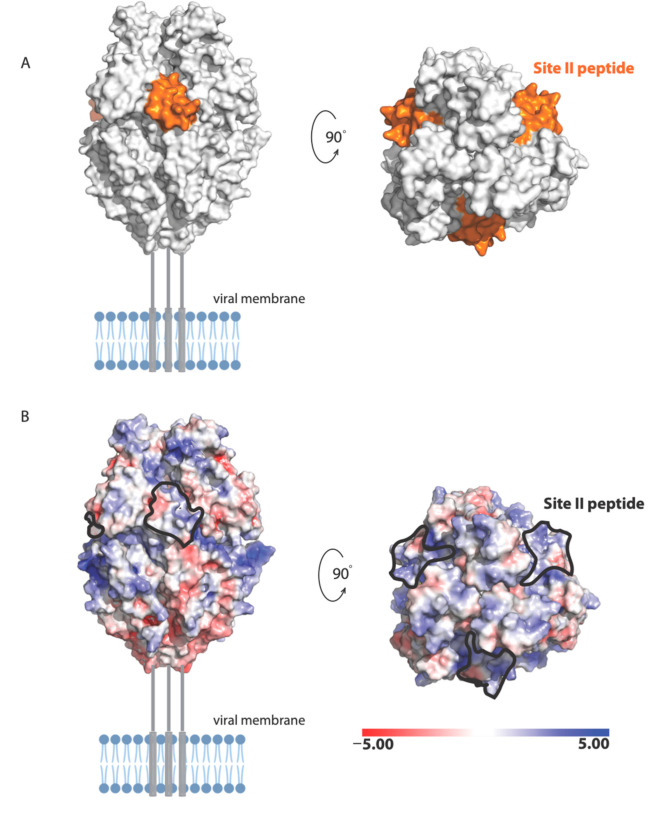
Illustration of the RSV-F trimer in the prefusion state (PDB code: 6VKD): (**A**) The site II peptide, highlighted in orange, may be important in RBD1,2 binding; (**B**) The electrostatic potential mapped onto the surface of RSV-F, with the location of site II epitope indicated with dotted lines. Electrostatic potential units are in KT/e. Missing regions are shown as grey lines.

Our results also suggest a potential mechanism by which Palivizumab (a humanized mouse monoclonal antibody used clinically in RSV prophylaxis [23]) neutralizes RSV infection, whereby Palivizumab binds RSV-F and blocks its interaction with nucleolin, which appears to be a necessary event for viral–cell fusion to occur. This is consistent with findings from other investigators who reported that Palivizumab does not block attachment or viral transcription, but most likely acts to inhibit viral fusion [24]. The antigenic site of RSV-F targeted by Palivizumab is NSELLSLINDMPITNDQKKLMSNN (also called site II [25]). The following residues in site II are surface exposed in the prefusion structure of RSV-F [19]: Asn254, Ser255, Glu256, Leu258, Ser259, Asn262, Asp263, Thr267, Asn268, Asp269, Lys271, Lys272, Ser275, Asn276, and Asn277 (Figure 8). These residues form a molecular surface with both positively and negatively charged patches (Figure 8A), suggesting that polar and electrostatic interactions may be important in the binding of RSV site II to nucleolin RBD1,2.

Analysis of the electrostatic potential of human nucleolin RBD1,2 reveals three patches of distinct electrostatic character: (a) strong positive, (b) polar and partial positive, and (c) negative (Figure 7D). Peptide A displays a polar and slightly positively charged surface. Peptide B is split into two surfaces with a strong positively charged patch at the bottom and a negatively charged surface at the top. The strong positively charged surface on peptide B is due to residues R347 and K348, which are likely important in RNA binding. Based on the physiochemical and electrostatic properties of the RSV site II, we hypothesize that the polar and partial positive surface from peptide A and negatively charged surface of peptide B are complementary to site II and the site of RSV-F glycoprotein engagement.

In conclusion, we have further characterized interactions between RSV and nucleolin, specifically RSV-F and RBD1,2, and identified domains on nucleolin that can be targeted for the development of novel compounds for RSV infections. Our results do not preclude that RSV-F may interact through other parts of the nucleolin protein, but suggest that the RBD1,2 domain interaction is critical for virus entry. Development of compounds that specifically bind and block viral interaction, with the region of nucleolin defined by peptides A and B, may be effective in RSV prophylaxis or therapy. Since nucleolin also has a role in the attachment and entry of a number of other viral pathogens, including influenza virus and HIV, these types of inhibitors may also be of value in a broader spectrum of viral infections.

## Figures and Tables

**Table 1:** Table of 12-mer peptides that react well with whole virus probe ^2^.

Spot # ^3^	Sequence	Amino Acid
A7 ^1^	TEPTTAFNLFVG	301–312
A8	TAFNLFVGNLNF	305–316
A18 ^1^	MTRKFGYVDFES	345–356
C9	EIDGRSISLYYT	452–464

^1^ Inhibits the virus in vitro. ^2^ All experiments to test peptide activity in virus assay were repeated 3 to 4 times with 6 replicates in each experiment. ^3^ “Spot#” refers to the position in the peptide array.

## References

[1] Pebody R., Moyes J., Hirve S., Campbell H., Jackson S., Moen A., Nair H., Simoes E.A.F., Smith P.G., Wairagkar N. (2019). Approaches to use the WHO respiratory syncytial virus surveillance platform to estimate disease burden. Influenza Other Respir. Viruses.

[2] Boyoglu-Barnum S., Chirkova T., Anderson L.J. (2019). Biology of Infection and Disease Pathogenesis to Guide RSV Vaccine Development. Front. Immunol..

[3] Servick K. (2020). Coronavirus creates a flu season guessing game. Science.

[4] Tayyari F., Marchant D., Moraes T.J., Duan W., Mastrangelo P., Hegele R.G. (2011). Identification of nucleolin as a cellular receptor for human respiratory syncytial virus. Nat. Med..

[5] Mastrangelo P., Norris M.J., Duan W., Barrett E.G., Moraes T.J., Hegele R.G. (2017). Targeting Host Cell Surface Nucleolin for RSV Therapy: Challenges and Opportunities. Vaccines (Basel).

[6] Griffiths C.D., Bilawchuk L.M., McDonough J.E., Jamieson K.C., Elawar F., Cen Y., Duan W., Lin C., Song H., Casanova J.L. (2020). IGF1R is an entry receptor for respiratory syncytial virus. Nature.

[7] Ginisty H., Sicard H., Roger B., Bouvet P. (1999). Structure and functions of nucleolin. J. Cell Sci..

[8] Jia W., Yao Z., Zhao J., Guan Q., Gao L. (2017). New perspectives of physiological and pathological functions of nucleolin (NCL). Life Sci..

[9] Su P.Y., Wang Y.F., Huang S.W., Lo Y.C., Wang Y.H., Wu S.R., Shieh D.B., Chen S.H., Wang J.R., Lai M.D. (2015). Cell surface nucleolin facilitates enterovirus 71 binding and infection. J. Virol..

[10] Nisole S., Said E.A., Mische C., Prevost M.C., Krust B., Bouvet P., Bianco A., Briand J.P., Hovanessian A.G. (2002). The anti-HIV pentameric pseudopeptide HB-19 binds the C-terminal end of nucleolin and prevents anchorage of virus particles in the plasma membrane of target cells. J. Biol. Chem..

[11] Chan C.M., Chu H., Zhang A.J., Leung L.H., Sze K.H., Kao R.Y., Chik K.K., To K.K., Chan J.F., Chen H. (2016). Hemagglutinin of influenza A virus binds specifically to cell surface nucleolin and plays a role in virus internalization. Virology.

[12] Bose S., Basu M., Banerjee A.K. (2004). Role of nucleolin in human parainfluenza virus type 3 infection of human lung epithelial cells. J. Virol..

[13] Techaarpornkul S., Collins P.L., Peeples M.E. (2002). Respiratory syncytial virus with the fusion protein as its only viral glycoprotein is less dependent on cellular glycosaminoglycans for attachment than complete virus. Virology.

[14] Johnson S.M., McNally B.A., Ioannidis I., Flano E., Teng M.N., Oomens A.G., Walsh E.E., Peeples M.E. (2015). Respiratory Syncytial Virus Uses CX3CR1 as a Receptor on Primary Human Airway Epithelial Cultures. PLoS Pathog..

[15] Kaan P.M., Hegele R.G. (2003). Interaction between respiratory syncytial virus and particulate matter in guinea pig alveolar macrophages. Am. J. Respir. Cell Mol. Biol..

[16] Keller G.A., Tokuyasu K.T., Dutton A.H., Singer S.J. (1984). An improved procedure for immunoelectron microscopy: Ultrathin plastic embedding of immunolabeled ultrathin frozen sections. Proc. Natl. Acad. Sci. USA.

[17] Ackerley C.A., Tilups A., Nielsen C., Coy M.A. (2003). Backscatter Electron Imaging Verses “Wein-type” Filtered Secondary Electron Imaging of Thick Methacrylate Sections of Tissues in a Field Emission Scanning Electron Microscope (FESEM). Microsc. Microanal..

[18] Arumugam S., Miller M.C., Maliekal J., Bates P.J., Trent J.O., Lane A.N. (2010). Solution structure of the RBD1,2 domains from human nucleolin. J. Biomol. NMR.

[19] McLellan J.S., Chen M., Leung S., Graepel K.W., Du X., Yang Y., Zhou T., Baxa U., Yasuda E., Beaumont T. (2013). Structure of RSV fusion glycoprotein trimer bound to a prefusion-specific neutralizing antibody. Science.

[20] Balcarova-Stander J., Pfeiffer S.E., Fuller S.D., Simons K. (1984). Development of cell surface polarity in the epithelial Madin-Darby canine kidney (MDCK) cell line. EMBO J..

[21] Anderson C.S., Chu C.Y., Wang Q., Mereness J.A., Ren Y., Donlon K., Bhattacharya S., Misra R.S., Walsh E.E., Pryhuber G.S. (2020). CX3CR1 as a respiratory syncytial virus receptor in pediatric human lung. Pediatr. Res..

[22] Chirkova T., Lin S., Oomens A.G.P., Gaston K.A., Boyoglu-Barnum S., Meng J., Stobart C.C., Cotton C.U., Hartert T.V., Moore M.L. (2015). CX3CR1 is an important surface molecule for respiratory syncytial virus infection in human airway epithelial cells. J. Gen. Virol..

[23] Null D., Bimle C., Weisman L., Johnson K., Steichen J., Singh S., Wang E., Asztalos E., Loeffler A.M., Azimi P.H. (1998). Palivizumab, a humanized respiratory syncytial virus monoclonal antibody, reduces hospitalization from respiratory syncytial virus infection in high-risk infants. Pediatrics.

[24] Huang K., Incognito L., Cheng X., Ulbrandt N.D., Wu H. (2010). Respiratory syncytial virus-neutralizing monoclonal antibodies motavizumab and palivizumab inhibit fusion. J. Virol..

[25] McLellan J.S., Chen M., Kim A., Yang Y., Graham B.S., Kwong P.D. (2010). Structural basis of respiratory syncytial virus neutralization by motavizumab. Nat. Struct. Mol. Biol..

